# ﻿Four new species of *Cryptothecia* (Arthoniaceae, Ascomycota) and *Myriostigma* (Arthoniaceae, Ascomycota) from China, based on morphology and molecular phylogeny

**DOI:** 10.3897/mycokeys.114.139180

**Published:** 2025-03-05

**Authors:** Junxia Xue, Zihao Yang, Ruotong Li, Lulu Zhang

**Affiliations:** 1 Institute of Environment and Ecology, Shandong Normal University, Jinan 250300, China Shandong Normal University Jinan China

**Keywords:** Diversity, identification key, Lichenized fungi, taxonomy

## Abstract

In this study, morphological and molecular phylogenetic approaches were used to investigate the species diversity of *Cryptothecia* and *Myriostigma* from southern China. We found four new species of *Cryptothecia* and *Myriostigma* (*C.disjecta*, *C.sorediatum*, *M.melanovillosa* and *M.visus-blotch*). *Cryptotheciadisjecta* is distinguished by verrucose pseudisidia that are loosely scattered on the thallus, the upper parts of which are white or have darker dots. *Cryptotheciasorediatum* is distinguished by I– medulla and thalli having globose soralia. *Myriostigmamelanovillosa* is distinguished by thalli with black or purple dots and hyaline to pale yellow ascospores (63–71 × 26–33 µm). *Myriostigmavisus-blotch* is distinguished by hyaline ascospores and ascigerous areas, which have many irregular small patches that are scattered or clustered together radially elongated. In addition, a phylogenetic tree based on mtSSU, RPB2 and nuLSU illustrates the phylogenetic placement of the proposed four new taxa. Detailed descriptions of the morphological, ecological and chemical characteristics and illustrations of each species are provided. Two updated keys for all known Chinese *Cryptothecia* and *Myriostigma* species are also provided.

## ﻿Introduction

Arthoniaceae (Arthoniales, Arthoniomycetes, Ascomycota) is widely distributed in tropical and subtropical regions. Arthoniaceae is characterized by its crustose, sometimes poorly developed or immersed, effuse or determinate thallus; trentepohlioid or rarely chlorococcoid photobiont; not determinate, often elongated and/or branched ascomata with rudimentary walls but rarely thick- and dark-walled; reddish or brownish, I+ blue hymenium; thick walled, ± fissitunicate, usually with a large apical dome and often I+ blue asci; septate and often multi-septate, occasionally muriform ascospores; and small, inconspicuous, coelomycetous (pycnidial) or sporodochial anamorph (Cannon et al. 2020). According to previous studies, the topology of the Arthoniaceae phylogenetic tree is mainly divided into Arthonioid and Cryptothecioid clades (Frisch et al. 2014; Thiyagaraja et al. 2020). *Cryptothecia*, *Herpothallon* and *Myriostigma* belong to the Cryptothecioid clade, and their morphological characteristics are similar, so distinguishing them is difficult. From 2020 to 2023, we collected a large number of lichen specimens from southern China while identifying a large number of species from these specimens. We found three new record species of *Cryptothecia*, eight new species and five new record species of *Herpothallon*, and three new species of *Myriostigma* (Chen et al. 2022; Liu et al. 2023a, 2023b; Xue et al. 2024; Zhang et al. 2024). Recently, we studied the remaining specimens, and we found that several of these specimens differed from the known species in their morphology and molecular phylogeny. Here, we describe two new species of *Cryptothecia* (*C.disjecta* and *C.sorediatum*) and two new species of *Myriostigma* (*M.melanovillosa* and *M.visus-blotch*).

## ﻿Materials and methods

### ﻿Morphology and anatomy

The study was based on specimens collected during fieldwork in Hainan and Yunnan Provinces, China, and preserved in the Lichen Section of Botanical Herbarium (SDNU, Shandong Normal University). Morphological features were studied with a dissecting microscope (COIC XTL7045B2), and photos were taken under a microscope (Olympus SZX16, Japan) with a DP72 camera system. The observations included the growth type, diameter and colour of the thallus; the shape and width of the prothallus; and the shape of the ascigerous areas. Anatomical features were observed and measured by a polarizing compound microscope (Olympus CX41, Japan), and images were taken under a microscope (Olympus BX61, Japan) with a DP72 camera system. The observations included the colour of the medulla and whether it contained crystals; the shape and size of the photobiont; the width of the hyphae; and the colour, shape and size of the asci, ascospores, pycnidia and conidia.

### ﻿Colour reaction and chemical analysis

Colour reactions were performed on the thallus surface and thin medulla sections with a saturated solution of aqueous sodium hypochlorite (C), a 10% aqueous solution of potassium hydroxide (K), a saturated solution of p-phenylenediamine in 95% ethyl alcohol (P), a 3% solution of Lugol’s iodine (I) and long-wavelength UV light. Polarized light microscopy (pol) was used to detect the presence of calcium oxalate crystals. The secondary metabolites of the lichens were analyzed and identified via thin layer chromatography (TLC) with solvent C (Orange et al. 2010; Elix 2014).

### ﻿DNA extraction, PCR amplification and sequencing

We extracted genomic DNA from the collected specimens using the Sigma-Aldrich REDExtract-N-Amp Plant PCR Kit (St. Louis, MO, USA) following the manufacturer’s protocol, except that only 30 μL of extraction buffer and 30 μL of dilution buffer were used. For extraction, the clean growing portions of the thalli of the specimens were selected under a dissecting microscope (COIC XTL7045B2) with sterile blades and forceps.

We amplified three gene sequences: the mtSSU gene with the primer pairs mtSSU1 and mtSSU3R (Zoller et al. 1999), the RPB2 gene with RPB2-7cF and RPB2-11aR (Liu et al. 1999), and the nuLSU gene with LIC24R and LR7 (Vilgalys and Hester 1990; Miadlikowska and Lutzoni 2000). We performed PCR amplification with a 50 μL volume containing 25 μL of 2 × Taq PCR MasterMix [Taq DNA Polymerase (0.1 unit/μL); 3 mM MgCl_2_; 100 mM KCl; 0.5 mM dNTPs; and 20 mM Tris-HCl (pH 8.3)] (Tiangen, Beijing, China), 19 μL of dd H_2_O, 2 μL of forward primer, 2 μL of reverse primer and 2 μL of DNA. The mtSSU gene was amplified via the following protocol: initial denaturation at 94 °C for 10 min; followed by 34 cycles at 95 °C for 45 s, 50 °C for 45 s, and 72 °C for 90 s; and a final extension at 72 °C for 10 min. The RPB2 gene was amplified via the following protocol: initial denaturation at 94 °C for 10 min; followed by 34 cycles at 94 °C for 45 s, 52 °C for 50 s, and 72 °C for 1 min; and a final extension at 72 °C for 5 min. The nuLSU gene was amplified via the following protocol: initial denaturation at 95 °C for 15 min; followed by 45 cycles at 95 °C for 45 s, 53 °C for 45 s, and 72 °C for 1 min; and a final extension at 72 °C for 7 min. PCR products were sequenced by BioSune Biological Technology (Shanghai, China) using the same primers.

### ﻿Sequence alignment and phylogenetic analysis

We compared the newly generated sequences with the available sequences in the GenBank database (http://www.ncbi.nlm.nih.gov/BLAST/) to ensure the reliability of all the new sequences. Then, we selected new sequences with high similarity to the clothing species of the *Cryptothecia* and *Myriostigma* for further analysis. We assembled the new sequences via SeqMan v.7.0 (DNAstar packages). The sequences of other genera of Arthoniaceae used in this study were downloaded from GenBank (Table 1). We aligned the sequences via the online version of MAFFT v.7.0.26. The algorithm of MAFFT is chosen automatically (FFT-NS-1, FFT-NS-2, FFT-NS-i or L-INS-i; depending on the data size). The sequences were edited via MEGA v.7.0. To construct the phylogenetic tree, the species *Chiodectonnatalense* Nyl. was selected as the outgroup taxon (Woo et al. 2017).

**Table 1.** Specimens used for the phylogenetic analyses with the corresponding voucher information and GenBank accession numbers for the mtSSU, RPB2 and nuLSU sequences. Newly obtained sequences in this study are in bold, * represents type material.

Species Name	Voucher Specimen	GenBank Accession Number		
		mtSSU	RPB2	nuLSU
*Arthoniacalcarea*	Thor 11/6a (UPS)	KJ850974	KJ851105	–
*Arthoniadidyma*	Ertz 7587 (BR)	EU704047	EU704010	EU704083
*Arthoniagranitophila*	Frish 10/Se74 (UPS)	KJ850981	KJ851107	KJ851049
*Arthoniagraphidicola*	Frisch 10/Jp102 (UPS)	KJ850980	–	KJ851034
*Arthoniailicina*	McCune 31067	KJ850982	–	–
*Arthoniaradiata*	Frisch 10/Se29 (UPS)	KJ850968	KJ851108	–
*Arthoniasubfuscicola*	Thor 11/1 (UPS)	KJ850971	KJ851110	–
*Arthotheliumruanum*	KoLRI 038018	MF616609	MF616619	–
*Arthotheliumruanum*	KoLRI 038261	MF616611	MF616621	–
*Arthotheliumspectabile*	Frisch 12Jp179a (TNS)	KP870144	KP870160	–
*Chiodectonnatalense*	Ertz 6576 (BR)	EU704051	EU704014	EU704085
*Coniocarponcinnabarinum*	Johnsen 111003 (UPS)	KJ850976	KJ851103	KJ851083
*Coniocarponfallax*	LD: L10075	KJ850979	KJ851101	–
*Crypthoniapalaeotropica*	Frisch 11/Ug457 (UPS)	KJ850961	KJ851084	–
*Cryptophaeaphaeospora*	Van den Broeck 5809 (BR)	KX077541	–	–
*Cryptotheciabartlettii*	Zhang et al. 20220297 (SDNU)	PP051262		PP583805
*Cryptotheciabartlettii*	Zhang et al. 20220275 (SDNU)	PP051261		PP583804
Cryptotheciadisjecta*	**Xue et al. 20230146 (SDNU)**	**PP587867**	–	–
***Cryptotheciadisjecta***	**Xue et al. 20230145 (SDNU)**	**PP587868**	–	–
*Cryptotheciainexspectata*	Liu et al. 20230668 (SDNU)	PP051263	PP109371	
*Cryptotheciainexspectata*	Liu et al. 20230639 (SDNU)	PP051264	PP109370	
*Cryptotheciastriata*	Liu et al. 20230938 (SDNU)	PP302048	PP585251	
*Cryptotheciastriata*	Liu et al. 20233925 (SDNU)	PP302049	PP585252	PP585252
Cryptotheciasorediatum*	**Liu et al. 20230379 (SDNU)**	**PP587866**	–	–
***Cryptotheciasorediatum***	**Liu et al. 20230381 (SDNU)**	**PP587865**	–	–
*Cryptotheciasubnidulans*	v.d.Boom 40613 (hd v.d. Boom)	KJ850952	KJ851087	–
*Cryptotheciasubnidulans*	Joensson Guyana 6a (UPS)	KJ850953	KJ851088	–
Glomerulophoronmauritiae*	Ertz 19164 (BR)	KP870153	KP870167	–
Herpothalloninopinatum*	Rudolphi 12 (UPS)	KJ850964	KJ851099	–
Herpothallonkigeziense*	Frisch 11/Ug26 (UPS)	KF707644	KF707654	–
*Herpothallonrubrocinctum*	Rudolphi 5 (UPS)	KF707643	KF707655	–
*Herpothallon* sp.	Frisch 11/Ug401 (UPS)	KF707645	KF707653	–
*Inodermabyssaceum*	Thor 25952 (UPS)	KJ850962	KJ851089	KJ851040
Inodermanipponicum*	Frisch 12Jp227 (TNS)	KP870146	KP870162	–
*Lepranthacinereopruinosa*	Kukwa 17127 & Lubek (BR)	MG207692	–	–
*Myriostigmacandidum*	Ertz 9260 (BR)	EU704052	EU704015	HQ454520
*Myriostigmacandidum*	Frisch 11/Ug125 (UPS)	KJ850959	KJ851096	–
Myriostigmaflavescens*	Liu et al. 20230612 (SDNU)	PP051268	PP130144	
*Myriostigmaflavescens*	Liu et al. 20230641 (SDNU)	PP051267		
Myriostigmahainana*	Xue et al. 20230061 (SDNU)	PP051271	PP101845	
*Myriostigmahainana*	Xue et al. 20230050 (SDNU)	PP051272	PP109365	
Myriostigmalaxipunctata*	Liu et al. 20231052 (SDNU)	PP051265	PP109368	PP033943
*Myriostigmalaxipunctata*	Liu et al. 20231231 (SDNU)	PP051266	PP109369	PP033944
Myriostigmamelanovillosa*	**Liu et al. 20230635 (SDNU)**	**PP587874**	**PP585246**	**PP583807**
***Myriostigmamelanovillosa***	**Liu et al. 20230629 (SDNU)**	**PP587875**	**PP585247**	–
Myriostigmaminiatum*	Silva T2A29 (ISE—epitype)	KP843606	–	–
Myriostigmavisus-blotch*	**Liu et al. 20231187 (SDNU)**	**PP587872**	**PP585249**	–
***Myriostigmavisus-blotch***	**Liu et al. 20230837 (SDNU)**	**PP587873**	**PP585248**	–
*Pachnolepiapruinata*	Frisch 11/Se34 (UPS)	KJ850967	KJ851098	–
*Reichlingialeopoldii*	Ertz 13293	JF830773	HQ454722	HQ454581
Reichlingiasyncesioides*	Frisch 11/Ug14 (UPS)	KF707651	KF707656	KF707636
Snippocianivea*	Ertz 17437 (BR)	MG207695	–	–
*Stirtonianeotropica*	Cáceres & Aptroot 11112 (ISE)	KP843611	–	–
Sporodophorongossypinum*	Frisch 12Jp186 (TNS)	KP870154	KP870168	–
Sporodophoronprimorskiense*	Ohmura 10509 (TNS)	KP870157	KP870169	–
Synarthoniaalbopruinosa*	VDB 6086 (BR<BEL>)	MH251873	–	–
*Synarthoniainconspicua*	VDB 7013B (BR<BEL>)	MH251881	–	–
*Synarthoniaochracea*	VDB 6653 (BR<BEL>)	MH251884	–	–
*Tylophorongalapagoense*	Bungartz 8749 (CDS)	JF830776	–	JF295078
*Tylophoronhibernicum*	Frisch 11/Ug220 (UPS)	KJ850966	KJ851097	KJ851065
*Tylophoronmoderatum*	Ertz 14504 (BR)	JF830780	–	JF295085

**Figure 1.**
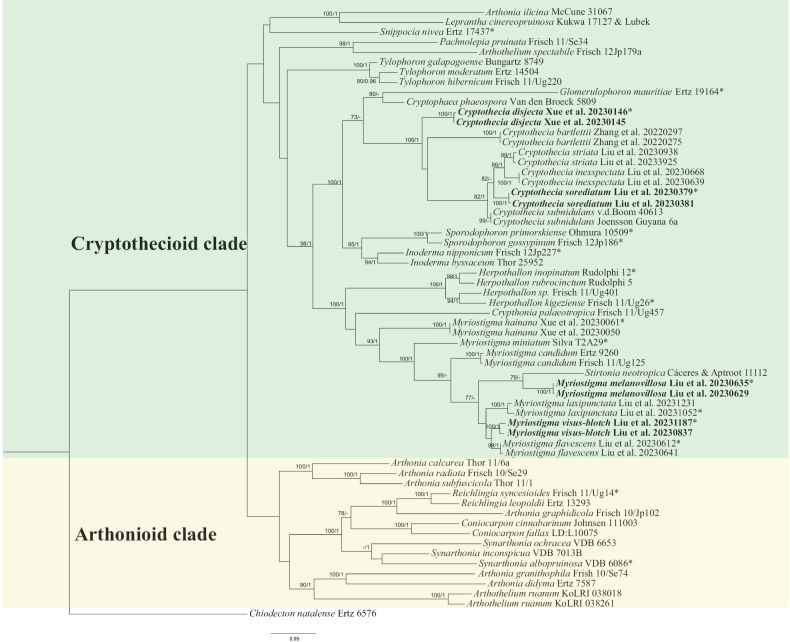
Phylogenetic tree constructed via maximum likelihood (ML) analysis of Arthoniaceae species on the basis of the concatenated mtSSU-RPB2-nuLSU dataset. Bootstrap support values ≥ 70 for ML and posterior probabilities ≥ 0.95 (second value) for Bayesian methods are indicated above or below the branches. Newly obtained sequences are marked in bold, * represents type material.

The multigene phylogenetic trees were inferred via maximum likelihood (ML) and Bayesian inference (BI). The three gene sequences were combined via the Concatenate Sequence function in PhyloSuite v1.2.3 (Zhang et al. 2020). We used the CIPRES Science Gateway (http://www.phylo.org/portal2/) (Miller et al. 2010) and performed ML analyses via RaxML-HPC v. 8.2.12 (Stamatakis 2014) under the default parameters implemented in CIPRES. Support values were based on 1000 nonparametric bootstrap pseudoreplicates. Bootstrap support values for ML equal to or greater than 70 were given above or below the nodes in the phylogenetic tree (Fig. 1). We used PhyloSuite to infer BI phylogenies via MrBayes 3.2.6 (Ronquist et al. 2012) under a partition model, for which the initial 25% of the sampled data were discarded as burn-in. Four Markov chains were run for 2,000,000 generations for the dataset. Trees were sampled every 1000^th^ generations. The stationarity of the analysis was determined by examining the standard deviation of the split frequencies (<0.01). Bayesian posterior probabilities equal to or greater than 0.95 were given above or below the nodes in the phylogenetic tree (Fig. 1). The phylogenetic trees generated were visualized via FigTree v1.4.2 (Rambaut 2012) and edited via Adobe Illustrator (AI). The new sequences were submitted to GenBank (Table 1).

## ﻿Results

### ﻿Phylogenetic analyses

A total of 8 mtSSU sequences, 4 RPB2 sequences and 1 nuLSU sequence were newly generated from 8 specimens. We constructed ML and BI topologies on the basis of these mtSSU, RPB2 and nuLSU sequences and 108 additional sequences downloaded from NCBI (https://www.ncbi.nlm.nih.gov/) (Table 1). The phylogenetic trees obtained from the ML and BI analyses presented similar topologies; therefore, we present only the ML tree, with BS ≥ 70 for the ML analysis and PP ≥ 0.95 for the Bayesian analysis (Fig. 1).

The phylogenetic tree structure we obtained was similar to that described in previous studies (Frisch et al. 2014; Thiyagaraja et al. 2020). All the species positions’ strongly supported the results of the ML and Bayesian analyses. Our phylogenetic tree revealed that *C.disjecta* and *C.sorediatum* clustered with *C.subnidulans* (the type species of *Cryptothecia*). However, on the basis of differences in phylogeny and morphology compared with those of *C.subnidulans*, which are described in detail below, they are classified as two new species. *M.melanovillosa* and *M.visus-blotch* clustered with *Myriostigma* and *Stirtonianeotropica* Aptroot. According to Aptroot (2009), *S.neotropica* has transversely septate ascospores; thus, on the basis of its morphological characteristics and phylogenetic analysis, we propose two new species in *Myriostigma*. Therefore, on the basis of morphological characteristics and phylogenetic analysis of the combined mtSSU, RPB2 and nuLSU sequence datasets, there is sufficient evidence to verify four previously unknown new species: *Cryptotheciadisjecta* J.X. Xue & Lu L. Zhang, sp. nov.; *C.sorediatum* J.X. Xue & Lu L. Zhang, sp. nov.; *Myriostigmamelanovillosa* J.X. Xue & Lu L. Zhang, sp. nov.; and *M.visus-blotch* J.X. Xue & Lu L. Zhang, sp. nov.

### ﻿Taxonomy

#### ﻿ Cryptothecia disjecta

Taxon classificationFungiArthonialesArthoniaceae

J.X. Xue & Lu L. Zhang sp. nov.

97D8EE49-011D-5432-ACDE-6A13A38A6173

855064

Fig. 2

##### Diagnosis.

The new species is distinguished from other *Cryptothecia* species by its verrucose pseudisidia, which are loosely scattered on the thallus. The upper parts of the pseudisidia are white or have darker dots.

##### Type.

China • Hainan Province, Baoting Li and Miao Autonomous County, Qixianling Hot Spring National Forest Park, 18°42'14.43"N, 109°41'47.92"E, alt. 325 m, on the bark of trees, 8 March 2023, J.X. Xue et al. 20230146 (SDNU, holotype).

##### Description.

***Thallus*** corticolous, up to 15 cm in diameter, ecorticate, cottony, dull, pale green, loosely attached to the substrate. ***Pseudisidia*** verrucose, loosely scattered on the thallus, upper parts white or with darker dots, and most pseudisidia have few projecting hyphae, 0.13–0.26 × 0.13–0.22 mm. ***Prothallus*** is usually distinct, thin, whitish byssoid, mainly composed of interwoven and radiating hyphae, 1.1–1.5 mm wide. ***Medulla*** white, with calcium oxalate crystals. ***Photobionts*** trentepohlioid, cells elliptical to oblong, single or aggregate into bundles, 10–23 × 7–18 µm. ***Hyphae*** 1–2.5 µm wide.

**Figure 2.**
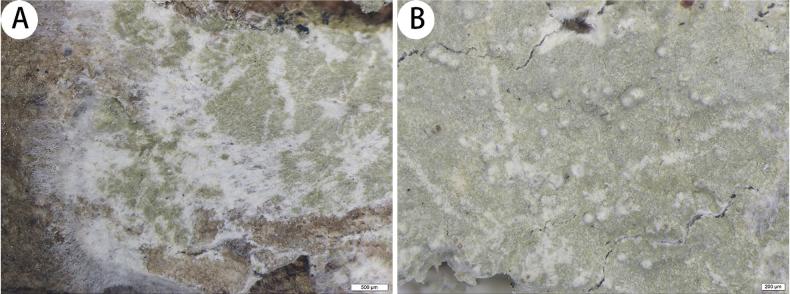
*Cryptotheciadisjecta* (SDNU 20230144, type) **A** thallus and prothallus **B** pseudisidia. Scale bars: 500 µm (**A**); 200 µm (**B**).

***Asci and pycnidia*** were not observed.

##### Chemistry.

thallus C+ red, K+ pale yellow, P–, UV+ pale grey-white; medulla and paraphysoids I+ sky-blue. TLC: gyrophoric acid and lecanoric acid.

##### Etymology.

The epithet refers to its pseudisidia, which are verrucose and loosely distributed across the thallus.

##### Ecology and distribution.

This species is found only in China on the bark of trees in a humid tropical forest in Hainan Province.

##### Notes.

Morphologically, *Cryptotheciadisjecta* is similar to both *Herpothallon* and *Cryptothecia.* However, we describe *C.disjecta* on the basis of its morphological and chemical characteristics and assign it to *Cryptothecia* on the basis of sequencing (Aptroot et al. 2024). *Cryptotheciadisjecta* is similar to *C.eungellae* G. Thor, as they both have whitish byssoid prothallus, I+ sky-blue medulla and C+ red thallus, but *C.eungellae* has 1-spored asci, muriform ascospores, and gyrophoric acid and norstictic acid as secondary metabolites (Thor 1997; Jagadeesh Ram and Sinha 2016).

Phylogenetically, *Cryptotheciadisjecta* is clustered with *C.bartlettii* G. Thor. They both have cottony thallus, whitish byssoid prothallus and C+ red thallus, but *C.bartlettii* has delimited ascigerous areas (developing in the thallus centre and covered with globose isidia-like structures), 1-spored asci and muriform ascospores (Thor 1997).

##### Additional specimens examined.

China • Hainan Province, Baoting Li and Miao Autonomous County, Qixianling Hot Spring National Forest Park, 18°42'14.43"N, 109°41'47.92"E, alt. 325 m, on the bark of trees, 8 March 2023, J.X. Xue et al. 20230144 (SDNU); • *ibid*., 20230145 (SDNU).

#### ﻿ Cryptothecia sorediatum

Taxon classificationFungiArthonialesArthoniaceae

J.X. Xue & Lu L. Zhang sp. nov.

73062017-1A15-5128-B1FB-098533134A32

855065

Fig. 3

##### Diagnosis.

The new species differs from other species of *Cryptothecia* in its soralia and I– medulla.

##### Type.

China • Yunnan Province, Xishuangbanna Dai Nationality Autonomous Prefecture, Jinghong City, Jinuo Mountain, Jinuo Ethnic Township, 21°54'52.26"N, 101°11'33.04"E, alt. 630 m, on the bark of trees, 3 March 2023, L.L. Liu et al. 20230379 (SDNU, holotype).

##### Description.

***Thallus*** corticolous, up to 4 cm in diameter, ecorticate, cottony, dull, pale green, firmly attached to the substrate. ***Soralia*** globose, with many calcium oxalate crystals, 0.07–0.2 × 0.07–0.2 µm. ***Prothallus*** is usually distinct, thick, whitish byssoid, mainly composed of interwoven and radiating hyphae, 0.9–1.7 mm wide. ***Medulla*** white, with calcium oxalate crystals. ***Photobionts*** trentepohlioid, cells rounded to elliptical, single or aggregate into bundles, 7–12 × 6–12 µm. ***Hyphae*** 1–2.5 µm wide.

**Figure 3.**
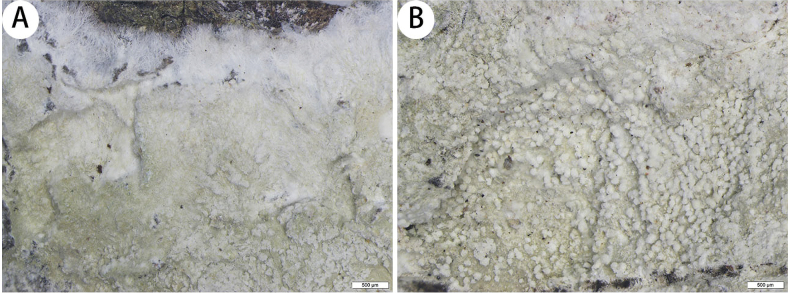
*Cryptotheciasorediatum* (SDNU 20230377, type) **A** thallus and prothallus **B** soralia. Scale bars: 500 µm (**A, B**).

***Asci and pycnidia*** were not observed.

##### Chemistry.

thallus C+ red, K–, P–, UV+ pale grey-white; medulla and paraphysoids I–. TLC: gyrophoric acid and lecanoric acid.

##### Etymology.

The epithet refers to the presence of soralia on its thallus.

##### Ecology and distribution.

This species is found only in China on the bark of trees in a humid tropical forest in Yunnan Province.

##### Notes.

Morphologically, *Cryptotheciasorediatum* is similar to *C.bartlettii* G. Thor, as they both have C+ red thallus and globose structures (*C.sorediatum* has globose soralia, and *C.bartlettii* has globose isidia-like structures) on their thallus, but *C.bartlettii* has I+ sky-blue medulla, 1-spored asci and muriform ascospores (Thor 1997).

Phylogenetically, *Cryptotheciasorediatum* is clustered with *C.subnidulans* Stirton, *C.inexspectata* G. Thor and *C.striata* G. Thor. They all have a cottony thallus and whitish byssoid prothallus, but *C.subnidulans* has a C– thallus, muriform ascospores (69–90 × 29–44 µm) and psoromic acid as a secondary metabolite (Thor 1997). *C.inexspectata* has whitish ascigerous areas, I+ sky-blue medulla and muriform ascospores (33–50 × 16–22 µm) (Thor 1997). *C.striata* has granular isidia-like structures on the thallus, I+ sky-blue medulla and muriform ascospores [(46–)55–70(–80) × (19–)23–29(–37) µm] (Thor 1991).

##### Additional specimens examined.

China • Yunnan Province, Xishuangbanna Dai Nationality Autonomous Prefecture, Jinghong City, Jinuo Mountain, Jinuo Ethnic Township, 21°54'52.26"N, 101°11'33.04"E, alt. 630 m, on the bark of trees, 3 March 2023, L.L. Liu et al. 20230377 (SDNU); • *ibid*., 20230381 (SDNU).

#### ﻿ Myriostigma melanovillosa

Taxon classificationFungiArthonialesArthoniaceae

J.X. Xue & Lu L. Zhang sp. nov.

91DCB7B4-BE04-5AE2-B50D-74C823DB5907

855066

Fig. 4

##### Diagnosis.

The new species differs from other species of *Myriostigma* in the presence of black or purple dots on the thalli and hyaline to pale yellow ascospores (63–71 × 26–33 µm).

##### Type.

China • Yunnan Province, Xishuangbanna Dai Nationality Autonomous Prefecture, Jinghong City, Primitive Forest Park, 22°1'55.75"N, 100°52'37.47"E, alt. 689 m, on the bark of trees, 7 March 2023, L.L. Liu et al. 20230635 (SDNU, holotype).

##### Description.

***Thallus*** corticolous, up to 6 cm in diameter, ecorticate, cottony, dull, greenish grey to whitish grey, with black or purple dots, firmly attached to the substrate. ***Isidia*** not observed. ***Prothallus*** is usually distinct, thin, whitish byssoid, mainly composed of interwoven and radiating hyphae, 0.7–1.3 mm wide, forming a dark brown to black line while bordering different species. ***Medulla*** white, with calcium oxalate crystals. ***Photobionts*** trentepohlioid, cells rounded to elliptical, single or a few cells aggregated, 5–11 × 5–8 µm. ***Hyphae*** 1–2 µm wide.

**Figure 4.**
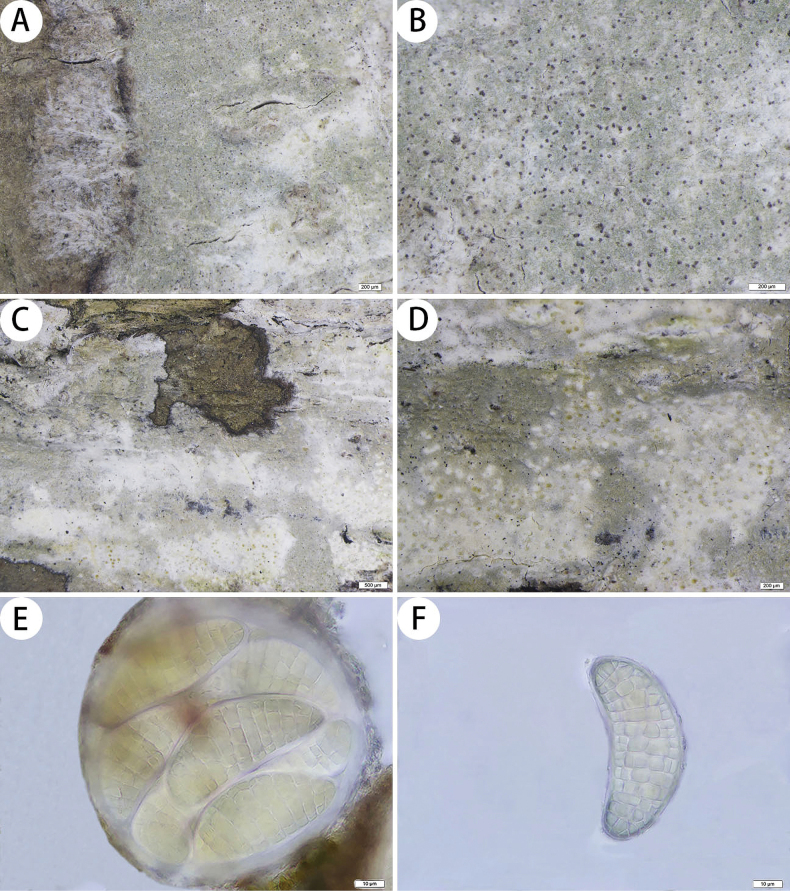
*Myriostigmamelanovillosa* (SDNU 20230629, type for (**A, B**); SDNU 20230635, holotype for (**C, D, E, F**)) **A** prothallus **B** black or purple dots **C** thallus **D** ascigerous areas **E** asci **F** ascospores. Scale bars: 200 µm (**A, B, D**); 500 µm (**C**); 10 µm (**E, F**).

***Ascigerous areas*** are distinct, generally delimited, erumpent, slightly raised above the thallus level, plaque, irregular in outline, white with dense brown dots indicating individual asci. ***Asci*** frequent, hyaline, pale yellow when mature, globose to subglobose, often covered by hyaline hyphae, 8-spored, 103–115 × 97–115 µm. ***Ascospores*** hyaline, pale yellow when mature, oblong, muriform, curved, often broader in the centre, 63–71 × 26–33 µm.

***Pycnidia*** were not observed.

##### Chemistry.

thallus C+ red, K–, P–, UV+ pale grey-white; medulla and paraphysoids I+ sky-blue. TLC: gyrophoric acid, lecanoric acid and confluentic acid.

##### Etymology.

The epithet refers to the presence of black or purple dots on the thallus.

##### Ecology and distribution.

This species is found only in China on the bark of trees in a humid tropical forest in Yunnan Province.

##### Notes.

Morphologically, *Myriostigmamelanovillosa* is similar to *M.irregularis* Lücking, Aptroot, Kalb & Elix, as they both have irregular erumpent and with brown dots whitish ascigerous areas, but *M.irregularis* has narrower asci (40–70 µm wide) and psoromic acid, subpsoromic acid, 2′-*O*-demethylpsoromic acid and trace confluentic acid as secondary metabolites (Lücking et al. 2006).

Phylogenetically, *Myriostigmamelanovillosa* is clustered with *Stirtonianeotropica* Aptroot. They both have C+ red thalli and 8-spored asci, but *S.neotropica* has linear shape ascigerous areas, ovoid asci and transversely septate ascospores (35–38 × 10–12 µm) (Aptroot 2009).

##### Additional specimens examined.

China • Yunnan Province, Xishuangbanna Dai Nationality Autonomous Prefecture, Jinghong City, Primitive Forest Park, 22°1'55.75"N, 100°52'37.47"E, alt. 689 m, on the bark of trees, 7 March 2023, L.L. Liu et al. 20230629 (SDNU); • *ibid*., 20234628 (SDNU).

#### ﻿ Myriostigma visus-blotch

Taxon classificationFungiArthonialesArthoniaceae

J.X. Xue & Lu L. Zhang sp. nov.

300734BC-667D-5854-9AD5-77EDF677CE68

855067

Fig. 5

##### Diagnosis.

The new species differs from other species of *Myriostigma* in ascigerous areas, which have many irregular small patches that are scattered or clustered together radially elongated; hyaline ascospores (31–)37–74 × (14–)17–29 µm.

##### Type.

China • Yunnan Province, Xishuangbanna Dai Nationality Autonomous Prefecture, Jinghong City, Primitive Forest Park, 22°2'9.71"N, 100°53'5.81"E, alt. 716 m, on the bark of trees, 7 March 2023, L.L. Liu et al. 20230681 (SDNU, holotype); • *ibid*., Mengla County, Menglun Town, Xishuangbanna Tropical Botanical Garden, 21°55'12.06"N, 101°16'5.55"E, alt. 496 m, on the bark of trees, 5 March 2023, L.L. Liu et al. 20231187 (SDNU, paratype).

**Figure 5.**
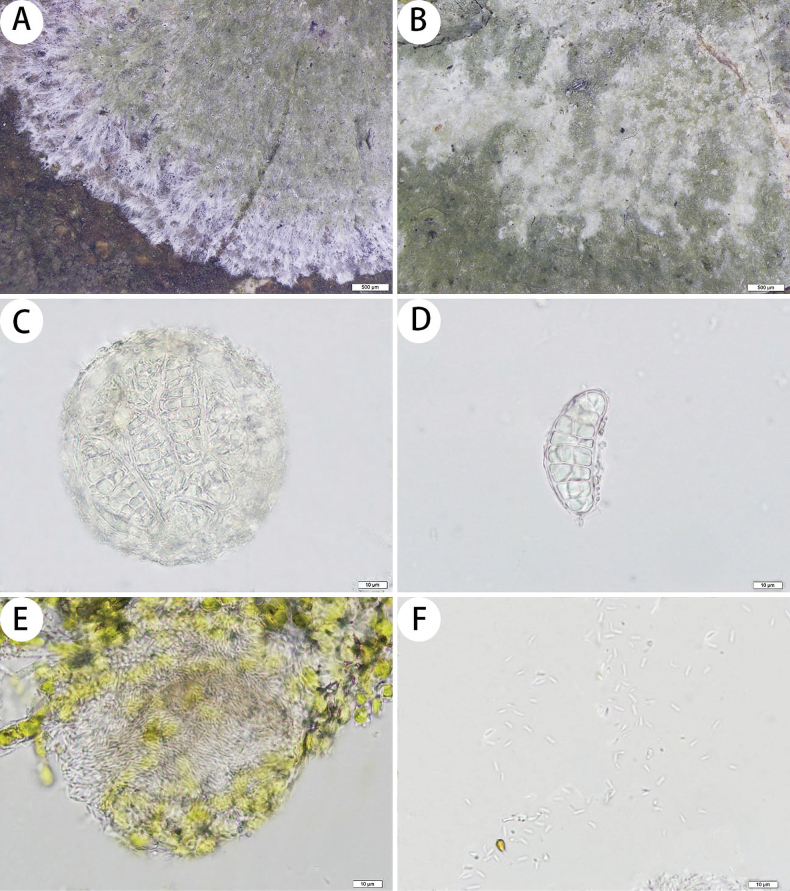
*Myriostigmavisus-blotch* (SDNU 20230950, type for (**A, B**); SDNU 20230681, holotype for (**C, D**); SDNU 20230847, type for (**E, F**)) **A** thallus and prothallus **B** ascigerous areas **C** asci **D** ascospores **E** pycnidia **F** conidia. Scale bars: 500 µm (**A, B**); 10 µm (**C, D, E, F**).

##### Description.

***Thallus*** corticolous or bambusicolous, up to 6.5 cm in diameter, ecorticate, cottony, dull, pale green to greenish grey, firmly attached to the substrate. ***Isidia*** not observed. ***Prothallus*** is usually distinct, thin, whitish byssoid, mainly composed of interwoven and radiating hyphae, 0.8–2.5 mm wide, forming a dark brown to black line while bordering different species. ***Medulla*** white, with calcium oxalate crystals. ***Photobionts*** trentepohlioid, cells elliptical to oblong, single or aggregate into bundles, 6–14 × 6–9 µm. ***Hyphae*** 1–2 µm wide.

***Ascigerous areas*** are distinct, generally delimited, slightly raised above the thallus level, developing in the thallus centre, colour lighter than the thallus, many irregular small patches that are scattered or clustered together radially elongated. ***Asci*** hyaline, globose to subglobose, often covered by hyaline hyphae, 8-spored, 82–91 × 81–90 µm. ***Ascospores*** hyaline, oblong, muriform, curved, often broader in the centre, (31–)37–74 × (14–)17–29 µm.

***Pycnidia*** hyaline, globose to subglobose, scattered and immersed across the thallus, 55–85 × 54–80 μm. ***Conidia*** hyaline, short bacilliform, 4–5 × 0.9–1 μm.

##### Chemistry.

thallus C+ red, K+ pale yellow, P–, UV+ pale grey-white; medulla and paraphysoids I+ sky-blue. TLC: gyrophoric acid, lecanoric acid and confluentic acid.

##### Etymology.

The epithet refers to its ascigerous areas having many irregular pale green small patches.

##### Ecology and distribution.

This species is found only in China on bamboo and trees in humid tropical forests in Hainan and Yunnan Provinces.

##### Notes.

*Myriostigmavisus-blotch* is widely distributed in the Xishuangbanna Dai Nationality Autonomous Prefecture. The ascigerous areas of *M.visus-blotch* begin as a small bulge slightly higher than the thallus and gradually become multiple small patches or radially elongated in the centre of the thallus, and the colour of the ascigerous areas is pale green. In addition, we found that species with asci generally do not have pycnidia, and species with pycnidia generally do not have asci.

Phylogenetically, *Myriostigmavisus-blotch* is clustered with *M.flavescens* J.X. Xue & Lu L. Zhang and *M.laxipunctata* J.X. Xue & Lu L. Zhang. They all have C+ red thalli, globose asci and muriform ascospores, but *M.flavescens* has a black linear shape prothallus, white ascigerous areas and yellow ascospores (58–76 × 19–28 µm). *M.laxipunctata* has complete ascigerous areas (not small patches or radially elongated), larger asci (95–124 × 93–119 µm), and loose brown dots indicating asci (Xue et al. 2024).

##### Additional specimens examined.

China • Hainan Province, Baoting Li and Miao Autonomous County, Qixianling Hot Spring National Forest Park, 18°42'14.43"N, 109°41'47.92"E, alt. 325 m, on the bark of trees, 8 March 2023, J.X. Xue et al. 20230142 (SDNU); • *ibid*., 20230138 (SDNU); • *ibid*., 20230134 (SDNU); • Yunnan Province, Xishuangbanna Dai Nationality Autonomous Prefecture, Jinghong City, Mengla County, Menglun Town, Xishuangbanna Tropical Botanical Garden, 21°55'45.40"N, 101°15'29.17"E, alt. 518 m, on the bark of trees, 4 March 2023, L.L. Liu et al. 20230950 (SDNU); • *ibid*., 20230969 (SDNU); • *ibid*., 21°55'30.14"N, 101°15'42.72"E, alt. 527 m, on the bark of trees, 5 March 2023, L.L. Liu et al. 20233946 (SDNU); • *ibid*., 21°55'50.99"N, 101°15'33.29"E, alt. 512 m, on the bark of trees, 4 March 2023, L.L. Liu et al. 20230973 (SDNU); • *ibid*., 21°55'55.96"N, 101°14'35.00"E, alt. 560 m, on the bark of trees, 4 March 2023, L.L. Liu et al. 20230847 (SDNU); • *ibid*., on bamboo, 4 March 2023, L.L. Liu et al. 20230837 (SDNU); • *ibid*., Jinuo Mountain, Jinuo Ethnic Township, 21°54'52.26"N, 101°11'33.04"E, alt. 640 m, on the bark of trees, 3 March 2023, L.L. Liu et al. 20230502 (SDNU); • *ibid*., Wild Elephant Valley, 22°10'37.70"N, 100°51'24.54"E, alt. 749 m, on the bark of trees, 6 March 2023, L.L. Liu et al. 20230602 (SDNU); • *ibid*., Primitive Forest Park, 22°2'9.71"N, 100°53'5.81"E, alt. 746 m, on the bark of trees, 7 March 2023, L.L. Liu et al. 20230692 (SDNU).

## ﻿Discussion

*Cryptothecia* was established in 1877 (Stirton 1877), and *Myriostigma* was established in 1874 (Krempelhuber 1874). These genera are prevalent in the humid tropical and subtropical forests of southern China, boasting a remarkable species diversity. Nevertheless, their morphological distinctions are scarce, making molecular analyses indispensable for classification and species identification. This study focused on Cryptothecioid lichens in Yunnan and Hainan Provinces of China, employing phylogenetic analyses based on DNA sequence data, and identified four novel species: *C.disjecta*, *C.sorediatum*, *M.melanovillosa*, and *M.visus-blotch*.

Prior to this study, China had reported eight species of *Cryptothecia* (*C.aleurella*, *C.aleurocarpa*, *C.bartlettii*, *C.inexspectata*, *C.polymorpha*, *C.striata*, *C.subnidulans*, and *C.subtecta*) and four species of *Myriostigma* (*M.candidum*, *M.flavescens*, *M.hainana*, and *M.laxipunctata*), as documented by Aptroot and Sipman (2001), Aptroot and Sparrius (2003), Aptroot and Rodrigues (2005), Xue et al. (2024), and Zhang et al. (2024). The two new *Cryptothecia* species, *C.disjecta* and *C.sorediatum*, are readily distinguishable from all other Chinese *Cryptothecia* species by their sterile thalli featuring verrucose pseudisidia (*C.disjecta*) or globose soralia (*C.sorediatum*), along with gyrophoric acid and lecanoric acid as secondary metabolites. Among the other Chinese *Cryptothecia* species containing gyrophoric acid, namely *C.bartlettii*, *C.inexspectata*, and *C.striata*, all are fertile (Thor 1997; Xue et al. 2024; Zhang et al. 2024). *Myriostigmamelanovillosa* is further distinguished from all other Chinese *Myriostigma* species by its thallus with black or purple dots, a whitish byssoid prothallus, and the presence of gyrophoric acid and confluentic acid. It also exhibits white plaque ascigerous areas with dense brown dots indicating individual asci, and hyaline to pale yellow ascospores. Although *M.flavescens* and *M.laxipunctata* also contain gyrophoric acid and confluentic acid, *M.flavescens* can be differentiated by its black line prothallus, smaller asci (95–100 × 83–95 µm), and yellow ascospores. Meanwhile, *M.laxipunctata* is distinguished by its pale greenish complete ascigerous area with loose brown dots (Xue et al. 2024). *M.visus-blotch* further distinguishes itself from all other Chinese *Myriostigma* species by its whitish byssoid prothallus, the presence of gyrophoric acid and confluentic acid, and pale greenish patchy (scattered or clustered together radially elongated) ascigerous areas. *Myriostigmahainana* also possesses a whitish byssoid prothallus and gyrophoric acid, but it can be differentiated by its indistinct ascigerous area, larger asci (120–138 × 120–135 µm), and the additional presence of methyl 2’-*O*-methylmicrophyllinate alongside gyrophoric acid (Xue et al. 2024).

In summary, the molecular phylogenetic and morphological results support the identification of the four new species in this study.

### ﻿Key to the *Cryptothecia* species occurring in China

**Table:** 

1	Thallus sterile; without asci	**2**
–	Thallus fertile; with asci	**3**
2	Thallus with verrucose pseudisidia; medulla I+ sky-blue	***C.disjecta***
–	Thallus with globose soralia; medulla I–	***C.sorediatum***
3	Asci 1–2-spored	**4**
–	Asci 8-spored	**7**
4	Thallus P+ yellow; with psoromic acid	***C.subnidulans***
–	Thallus P–; without psoromic acid	**5**
5	Thallus loosely attached to the substrate; ascigerous areas generally covered with globose isidia-like structures; ascospores (49–)68–100(–105) × (18–)23–36(–42) µm	***C.bartlettii***
–	Thallus firmly attached to the substrate; ascigerous areas whitish and usually radially elongated	**6**
6	Thallus generally with granula isidia-like structures; ascospores 54–80 × 21–42 µm	***C.striata***
–	Thallus generally without isidia-like structures; ascospores 33–50 × 16–22 µm	***C.inexspectata***
7	Thallus without lichen substances	**8**
–	Thallus with lichen substances	**9**
8	Ascospores narrow; 60–76 × 17–30 µm	***C.aleurella***
–	Ascospores broad; 65–108 × 42–50 µm	***C.aleurocarpa***
9	Thallus P+ yellow; with psoromic acid; ascospores 50–70 × 30–37 µm	***C.polymorpha***
–	Thallus P–; without psoromic acid; ascospores 27–40 × 12–20 µm	***C.subtecta***

### ﻿Key to the *Myriostigma* species occurring in China

**Table:** 

1	Ascigerous areas indistinct; thallus with gyrophoric acid and methyl 2’-*O*-methylmicrophyllinate; ascospores 52–88 × 24–47 µm	***M.hainana***
–	Ascigerous areas distinct	**2**
2	Thallus with 2’-*O*-methylanziaic and 2’-*O*-methylperlatolic acids; ascospores 40–65 × 12–25 µm	***M.candidum***
–	Thallus with gyrophoric and confluentic acids	**3**
3	Ascigerous areas without brown dots indicate individual asci; ascospores hyaline (31–)37–74 × (14–)17–29 µm; some specimens have pycnidia	***M.visus-blotch***
–	Ascigerous areas with brown dots indicate individual asci	**4**
4	Thallus with black or purple dots; ascospores hyaline to pale yellow 63–71 × 26–33 µm	***M.melanovillosa***
–	Thallus without black or purple dots	**5**
5	Ascigerous areas with dense brown dots; ascospores yellow 58–76 × 19–28 µm	***M.flavescens***
–	Ascigerous areas with loosely brown dots; ascospores hyaline 57–78 × 24–33 µm	***M.laxipunctata***

## Supplementary Material

XML Treatment for Cryptothecia disjecta

XML Treatment for Cryptothecia sorediatum

XML Treatment for Myriostigma melanovillosa

XML Treatment for Myriostigma visus-blotch

## ﻿Additional information

### Author ORCIDs

Junxia Xue https://orcid.org/0000-0002-6387-0148

Zihao Yang https://orcid.org/0009-0001-0699-2229

Ruotong Li https://orcid.org/0009-0003-9428-3055

Lulu Zhang https://orcid.org/0000-0001-8011-4451

### Data availability

All of the data that support the findings of this study are available in the main text.

## References

[B1] Aptroot A (2009) A revision of the lichen genus *Stirtonia*. Lichenologist 41(5–6): 1–11. 10.1017/S0024282909990107

[B2] Aptroot A Rodrigues AF (2005) New lichen records for the Azores, with the report of some tropical species new to Europe. Cryptogamie. Mycologie 26(3): 273–280.

[B3] Aptroot A Sipman HJM (2001) New Hong Kong Lichens, Ascomycetes and lichenicolous fungi. The Journal of the Hattori Botanical Laboratory 91: 317–343.

[B4] Aptroot A Sparrius LB (2003) New microlichens from Taiwan. Fungal Diversity 14(1): 1–50.

[B5] Aptroot A Cáceres MES dos Santos LA (2024) The taxonomy of sterile Arthoniaceae from Brazil: White crusts on overhanging tropical trees can be named. Lichenologist 56(1): 1–13. 10.1017/S0024282924000021

[B6] Cannon PF Aptroot A Coppins BJ Orange A Sanderson NA Simkin JA Yahr R (2020) Revisions of British and Irish Lichens. British Lichen Society, London, 48 pp.

[B7] Chen PF Liu LL Xie CM Zhang L (2022) Four new species of *Herpothallon* (Arthoniaceae, Arthoniales, Arthoniomycetes, Ascomycota) from China. Phytotaxa 536(1): 83–91. 10.11646/phytotaxa.536.1.5

[B8] Elix JA (2014) A Catalogue of Standardized Thin-Layer Chromatographic Data and Biosynthetic Relationships for Lichen Substances, 3^rd^ edn. Australian National University, Canberra.

[B9] Frisch A Thor G Ertz D Grube M (2014) The Arthonialean challenge: Restructuring Arthoniaceae. Taxon 63(4): 727–744. 10.12705/634.20

[B10] Jagadeesh Ram TAM Sinha GP (2016) A world key to *Cryptothecia* and *Myriostigma* (Arthoniaceae), with new species and new records from the Andaman and Nicobar Islands, India. Phytotaxa 266(2): 103–114. 10.11646/phytotaxa.266.2.4

[B11] Krempelhuber A (1874) Lichenes foliicolae quos legit O. Beccari annis 1866–1867 in insula Borneo. Privately published, München, without pagination.

[B12] Liu YJ Whelen S Hall BD (1999) Phylogenetic relationships among ascomycetes: Evidence from an RNA polymerase II subunit. Molecular Biology and Evolution 16(12): 1799–1808. 10.1093/oxfordjournals.molbev.a026092 10605121

[B13] Liu LL Zuo QJ Zhang LL (2023a) Species diversity and floristic elements of the lichen genus *Herpothallon* in China. Guihaia 43(7): 1268–1275.

[B14] Liu LL Zuo QJ Xue JX Ren ZJ Zhang LL (2023b) Three new species of *Herpothallon* (Lichenized Ascomycota) from Southern China. Phytotaxa 597(4): 287–296. 10.11646/phytotaxa.597.4.4

[B15] Lücking R Thor G Aptroot A Kalb K Elix JA (2006) The *Cryptotheciacandida* complex revisited. Lichenologist 38(3): 235–240. 10.1017/S0024282906005603

[B16] Miadlikowska J Lutzoni F (2000) Phylogenetic revision of the genus *Peltigera* (lichen-forming ascomycetes) based on morphological, chemical and large subunit nuclear ribosomal DNA data. International Journal of Plant Sciences 161(6): 925–985. 10.1086/317568

[B17] Miller MA Pfeiffer W Schwartz T (2010) Creating the CIPRES Science Gateway for inference of large phylogenetic trees. In: Proceedings of the Gateway Computing Environments Workshop (GCE), New Orleans, LA, 14 November 2010. 10.1109/GCE.2010.5676129

[B18] Orange A James PW White FJ (2010) Microchemical Methods for the Identification of Lichens, 2^nd^ edn. British Lichen Society, London, 101 pp.

[B19] Rambaut A (2012) FigTree, v. 1.4.0. Institute of Evolutionary Biology, University of Edinburgh, 2012. http://tree.bio.ed.ac.uk/software/Figtree/

[B20] Ronquist F Teslenko M Van Der Mark P Ayres DL Darling A Höhna S Larget B Liu L Suchard MA Huelsenbeck JP (2012) MrBayes 3.2: Efficient Bayesian phylogenetic inference and model choice across a large model space. Systematic Biology 61(3): 539–542. 10.1093/sysbio/sys029 22357727 PMC3329765

[B21] Stamatakis A (2014) RaxML version 8: A tool for phylogenetic analysis and postanalysis of large phylogenies. Bioinformatics 30(9): 1312–1313. 10.1093/bioinformatics/btu033 24451623 PMC3998144

[B22] Stirton J (1877) Description of recently discovered foreign lichens. Proceedings of the Royal Philosophical Society of Glasgow 10: 156–164.

[B23] Thiyagaraja V Lücking R Ertz D Wanasinghe DN Karunarathna SC Camporesi E Hyde KD (2020) Evolution of non-lichenized, saprotrophic species of *Arthonia* (Ascomycota, Arthoniales) and resurrection of *Naevia*, with notes on *Mycoporum*. Fungal Diversity 102(11): 205–224. 10.1007/s13225-020-00451-9

[B24] Thor G (1991) The Placement of *Chiodectonsanguineum* (syn. *Chiodectonrubrocinctum*), and *Cryptotheciastriata* sp. nov. The Bryologist 94(3): 278–283. 10.2307/3243965

[B25] Thor G (1997) The genus *Cryptothecia* in Australia and New Zealand and the circumscription of the genus. Symbolae Botanicae Upsalienses 32(1): 267–289.

[B26] Vilgalys R Hester M (1990) Rapid genetic identification and mapping of enzymatically amplified ribosomal DNA from several *Cryptococcus* species. Journal of Bacteriology 172(8): 4238–4246. 10.1128/jb.172.8.4238-4246.1990 2376561 PMC213247

[B27] Woo JJ Lőkös L Farkas E Park C-H Hur J-S (2017) *Cryptotheciaaustrocoreana* (Arthoniales, Arthoniaceae), a New Species from South Korea. Mycobiology 45(4): 338–343. 10.5941/MYCO.2017.45.4.338 29371801 PMC5780365

[B28] Xue JX Cai YT Zhang LL (2024) The phylogeny and taxonomy of *Cryptothecia* (Arthoniaceae, Ascomycota) and *Myriostigma* (Arthoniaceae, Ascomycota), including three new species and two new records from China. Journal of Fungi 10(4): 274–288. 10.3390/jof10040274 38667945 PMC11051358

[B29] Zhang D Gao F Jakovlić I Zou H Zhang J Li WX Wang GT (2020) PhyloSuite: An integrated and scalable desktop platform for streamlined molecular sequence data management and evolutionary phylogenetics studies. Molecular Ecology Resources 20(1): 348–355. 10.1111/1755-0998.13096 31599058

[B30] Zhang LL Xue JX Liu LL (2024) A new species and a new record of byssoid Arthoniaceae (Lichenized Ascomycota) from southern China. Diversity 16(5): 287–295. 10.3390/d16050287

[B31] Zoller S Scheidegger C Sperisen C (1999) PCR primers for the amplification of mitochondrial small subunit ribosomal DNA of lichen-forming ascomycetes. Lichenologist 31(5): 11–516. 10.1006/lich.1999.0220

